# The clinical efficacy of novel semirigid ureteroscopy with a vacuum suction device for treatment of >10 mm upper ureteral stones and impacted calculus: implications for clinical practice

**DOI:** 10.3389/fsurg.2025.1656819

**Published:** 2026-01-02

**Authors:** Longhui Lai, Yuanfeng Zhang, Yingru Wang, Enguang Yang, Guangrui Fan, Chaohu Chen, Kang Yao, Wenzhao Zhang, Yongfei Liu, Kai Zhu, Meixuan Ding, Jinchun Xing, Zhiping Wang, Peide Bai, Tao Wang, Bin Chen, Jun Mi

**Affiliations:** 1 Department of Urology, The Second Hospital of Lanzhou University, The Second Clinical Medical School, Gansu Province Clinical Research Center for Urinary System Disease, Lanzhou, China; 2 The Key Laboratory of Urinary Tract Tumors and Calculi, Department of Urology Surgery, The First Affiliated Hospital, School of Medicine, Xiamen University, Xiamen, China; 3 Department of Urology, Shantou Central Hospital, Shantou, China; 4 Department of Pediatric Surgery, Women and Children’s Hospital, School of Medicine, Xiamen University, Xiamen, China; 5 Department of Urology, Longyan First Affiliated Hospital of Fujian Medical University, Longyan, China; 6 Medical Team of the Zhengzhou Detachment, Henan Provincial Corps, Chinese People's Armed Police Force, Zhengzhou, China; 7 The School of Clinical Medicine, Fujian Medical University, Fuzhou, China

**Keywords:** upper ureteral stone, impacted calculi, semirigid ureteroscopy, vacuum suction device, lithotripsy procedures

## Abstract

**Objective:**

The novel semirigid ureteroscopy with a vacuum suction device has been increasing in the treatment of urinary stones recently. This study aimed to evaluate its clinical efficacy for treating >10 mm upper ureteral stones and impacted calculus and to further assess the efficacy by using a vacuum suction device during lithotripsy procedures.

**Patients and methods:**

This study included 156 patients with >10 mm upper ureteral calculi who underwent laser lithotripsy under the novel semirigid ureteroscopy with a vacuum suction device. The vacuum suction device and the end of the ureteral access sheath (UAS) were joined together to create a closed circulation working channel between the former and the collection system. This circulation working channel allows the operator to suction stone fragments out of the body during lithotripsy. Based on impacted and non-impacted upper ureteral stones, subgroup analysis was performed.

**Results:**

Of the 156 patients, 150 underwent successful phase I ureteral sheath placement and lithotripsy. 6 patients were unsuccessfully placed for UAS because of upper ureteral stenosis, but all of them received indwelling Double-J tubes for one month and then underwent successful second-stage lithotripsy. The average operative time was 43.8 ± 25.7 min. The stone-free rate (SFR) was 57.7% (90/156) at 1 day postoperatively, and 87.8% (137/156) at 1 month postoperatively. The rate of complications was 9.0% (14/156). In subgroup analysis, for the impacted stone group, the average operative time was 48.0 ± 29.3 min. The SFR was 54.8% (46/84) at 1 day postoperatively, and 88.1% (74/84) at 1 month postoperatively. The rate of complications was 10.7% (9/84).

**Conclusion:**

For the treatment of >10 mm upper ureteral stones and impacted calculus, the novel semirigid ureteroscopy with a vacuum suction device is safe and effective. During the lithotripsy procedure, the use of a vacuum suction device has the potential to improve stone removal efficiency. However, due to the critical limitation of the lack of integrated pressure monitoring and control devices, potential complications caused by negative pressure suction and perfusion flow imbalance should be watched out for.

## Introduction

Urinary stone disease is a prevalent global urological condition with a steadily increasing incidence (1, 2). Upper urinary tract stones (including kidney stones and ureteral stones) constitute the predominant component of urinary stones, accounting for over 90% of cases; among these, ureteral stones represent approximately 30%–40% of all urinary stone cases (3, 4). The clinical presentation of upper ureteral stones often includes unilateral colicky pain that can significantly impair quality of life and may result in death in the worst cases (5). There are no international guidelines defining larger proximal ureteral stones, but there are isolated reports in the literature defining stones larger than 10 mm as larger proximal ureteral stones (6, 7). An impacted ureteral calculus is usually defined as a stone that has stayed in the same location in the ureter for at least two months, is unable to pass out on its own, and causes ureteral obstruction (8).

Currently, ureteroscopic lithotripsy (URS) is widely recommended for the management of mid-to-upper ureteral stones (9). However, conventional rigid ureteroscopic lithotripsy (R-URS) is associated with a significantly lower SFR and a higher risk of stone migration when treating larger upper ureteral stones (10). Although flexible ureteroscopic lithotripsy (F-URS) offers satisfactory efficacy and is considered a first-line option for upper ureteral stones, its use is limited by high costs, inferior durability, and expensive maintenance (11, 12). Moreover, particularly in elderly patients, F-URS for larger proximal ureteral stones may result in suboptimal SFR and an increased risk of complications such as severe infections and stone bit formation (13).

Recently, a novel semirigid ureteroscope integrated with a vacuum suction device was developed in China (14). It integrates the benefits of vacuum suction and perfusion in the UAS to achieve simultaneous lithotripsy and stone removal, lower intra-renal pressure, and clear surgical fields. Preliminary studies have demonstrated the safety and efficacy of the novel semirigid ureteroscopy with a vacuum suction device for treating renal pelvic and proximal ureteral stones (14, 15).

Based on the “simultaneous lithotripsy and stone removal” feature of novel vacuum suction semirigid ureteroscopy, it may theoretically have an advantage in the treatment of larger upper ureteral stones and impacted calculus. However, clinical evidence validating its superior efficacy and efficiency in such cases remains limited. Therefore, this study aimed to retrospectively evaluate the clinical efficacy of the novel vacuum suction semirigid ureteroscopy in treating upper ureteral stones larger than 10 mm and impacted calculi and further assess the efficacy by using a vacuum suction device during lithotripsy procedures.

## Patients and methods

### The patients

Following ethical approval from the Ethics Committee of the First Affiliated Hospital of Xiamen University (Grant Number: KY-064-01), a retrospective analysis was conducted on patients who underwent treatment for unilateral upper ureteral calculi larger than 10 mm using the novel vacuum suction semirigid ureteroscopy at our institution between January 2018 and December 2022. Inclusion criteria: (1) The patients' ages varied from 18 to 80 years old. (2) Patients were informed and consented to the study. (3) The stone was located between the renal pelvic-ureteral junction and the lower edge of the L4 spine, and the maximum diameter of the stone was >10 mm on a urinary tract CT(CT cuts were 3 mm). Exclusion criteria: (1) Patients with kidney stones, bladder stones, or bilateral ureteral stones. (2) Anatomical abnormalities of the urinary tract. (3) Abnormal cardiopulmonary function, unable to tolerate surgery. (4) Coagulation abnormalities. (5) Active infection of the urinary tract. This study only included cases that were successfully completed using the novel vacuum suction semirigid ureteroscopy alone. According to the aforementioned criteria, a total of 156 patients were enrolled in this study.

### The novel vacuum suction semirigid ureteroscopy device

**Figure 1:**
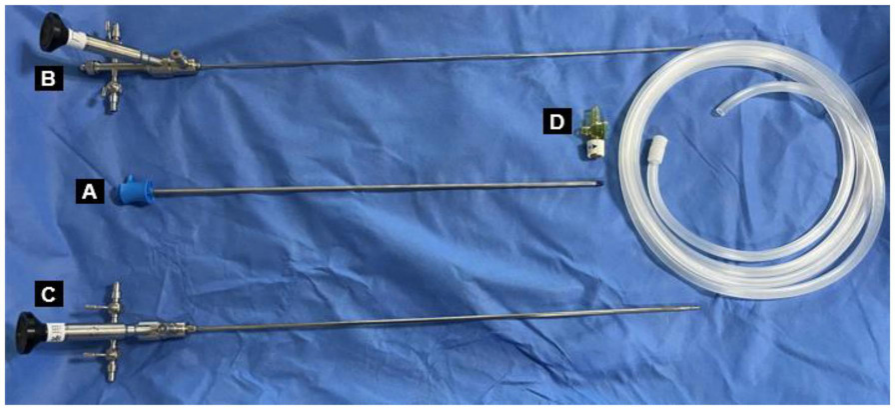
Components of the novel vacuum suction semirigid ureteroscopy: **(A)** The rigid ureteral access sheath. **(B)** Standard ureteroscope. **(C)** Lithotripsy ureteroscope. **(D)** Vacuum suction controller.

**Figure 2:**
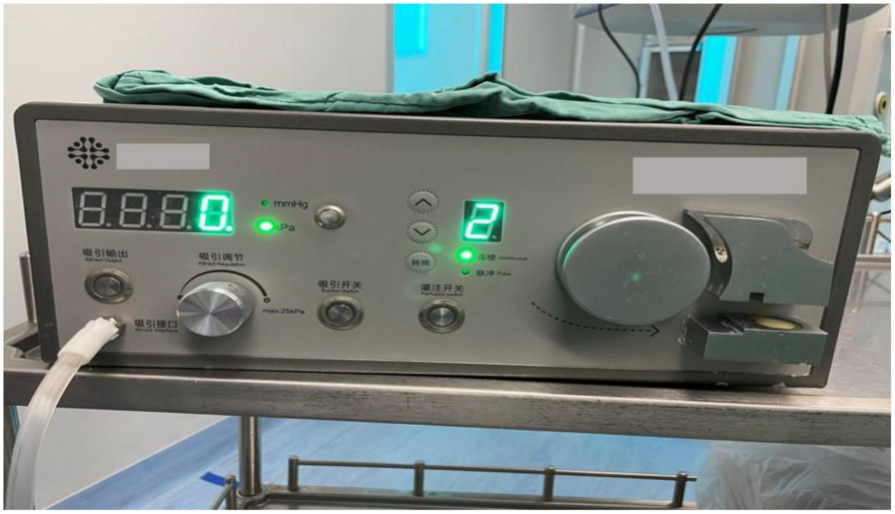
Negative pressure irrigation suction device.

The novel vacuum suction semirigid ureteroscopy (Shuo Tong Medical, Jiangmen, China) consists of the following four components: a rigid ureteral access sheath (F11.5/13.5), a standard ureteroscope (F7.5/11.5), a lithotripsy ureteroscope (F4.5/6.5), a negative pressure irrigation suction device (Figures 1, 2).

### Surgical procedure

After being given general anesthesia, the patient was placed in the standard lithotomy position. After attaching the rigid UAS and standard ureteroscope, the surgeon carefully inserted the above-mentioned combination into the renal pelvic-ureteral junction or beneath the stone's location under direct vision with the help of a guidewire (0.888 mm × 150 cm, C.R. Bard Inc., Murray Hill, NJ, USA) and then exited the standard ureteroscope. Subsequently, the negative pressure irrigation suction device and the end of the UAS were joined together to create a closed circulation working channel between the former and the collection system. The lithotripsy ureteroscope and a 200 um holmium laser fiber (Lumenis, Beijing, China) were inserted via the UAS into the stone site for dusting the stones. The surgical operator repeatedly withdrew the lithotripsy ureteroscope into the end of the UAS to suction out larger stone fragments (diameter: about 4.0 mm) during lithotripsy. The surgical operator adjusted the rotary button for negative pressure in real-time to control the force of the negative pressure attraction and prevent stones from moving upward into the renal pelvis. After completing the lithotripsy, the surgeon used the standard ureteroscope in place of the lithotripsy scope and then carefully removed the UAS and the standard ureteroscope under direct vision, observing the renal pelvis and ureteral mucosa for any damage. At the end of the procedure, 7F Double-J tubes and an F20 three-chamber catheter were routinely inserted. The Double-J tubes were placed for a duration of four weeks postoperatively and removed at the one-month follow-up. For stones firmly adherent to the ureteral wall and causing stenosis, patients underwent a second-stage procedure with the novel vacuum suction semirigid ureteroscope lithotripsy after four weeks of Double-J stent placement.

All surgeries were performed by a dedicated team of experienced endourologists. The surgical team consisted of two experienced chief urologists, each of whom had independently performed over 5,000 cases of conventional ureteroscopic lithotripsy and had completed a structured training on the novel vacuum suction semirigid ureteroscopy prior to the commencement of this study. A standardized surgical protocol, as detailed in the “Surgical procedure” section, was strictly followed by all surgeons to minimize technique-related variations. Key procedural steps, including the technique for UAS placement under direct vision, laser settings, and the dynamic adjustment of negative pressure suction to prevent stone migration, were uniformly applied across all cases. This approach was implemented to reduce individual operator bias and to enhance the consistency and reproducibility of our findings. All references to ureteroscopy in the manuscript were to the primary procedure. All patients with positive preoperative urine cultures were offered sensitive antibiotic therapy for 4–7 days before the surgical intervention. Patients with negative urine cultures received standard perioperative antibiotic prophylaxis. The power settings of the holmium lasers were 0.3–0.8J/20–30 Hz. In order to evaluate stone clearance, patients were examined by urinary tract CT (CT cuts were 3 mm) at one day and one month postoperatively. SFR was defined as no stone fragments remaining or fragments with a diameter <4 mm on CT scan (16, 17). The operative time was defined as the duration of anesthesia from the beginning to the end. The average postoperative follow-up time for the patients was six months to assess long-term complications such as ureteral stricture. Complications were assessed using the modified Clavien-Dindo grading system. Postoperative fever was defined as a temperature >38 °C for >48 h (18).

Given that patients with impacted ureteral stones are at high risk for postoperative ureteral stricture, this study implemented a strict, regular follow-up protocol for these patients. These patients underwent systematic evaluations at three key time points—1, 3, and 6 months postoperatively—using examinations such as urinary system ultrasonography and computed tomography urography (CTU), with the aim of timely detection and management of long-term complications like ureteral stricture.

### Statistical methods

The statistical analysis was executed utilizing SPSS 23.0 software (IBM SPSS; Armonk, NY, USA). Measurement data were displayed as means ± SD. Counting data were described as percentages (%). Continuous data with a normal distribution were submitted to the student's *t*-test, whereas continuous data with a nonnormal distribution were subjected to the Mann–Whitney rank-sum test. *P*-values < 0.05 were considered an indicator of a statistically significant difference.

## Results

### Main group analysis

During the data collection phase (168 cases), a total of 12 patients underwent combined the novel vacuum suction semirigid ureteroscopy with flexible ureteroscopy lithotripsy; however, in accordance with the study protocol, cases involving this combined procedure were excluded from the final analysis. Ultimately, all 156 patients meeting this research protocol underwent successful surgeries, and Table 1 exhibited the clinical baseline data for the patients. Six patients were unsuccessfully placed for UAS because of upper ureteral stenosis, but all of them received indwelling Double-J tubes for four weeks and then underwent successful second-stage lithotripsy. No ureteral strictures were discovered during the postoperative follow-up period.

**Table 1:** Main group analysis: classifications of preoperative baseline data, intraoperative data, postoperative data and surgical complications.

Variables	Value
Patients (*n*)	156
Age, years (mean ± SD)	49.8 ± 12.4
BMI, kg/m^2^ (mean ± SD)	24.6 ± 3.0
Gender (*n*)	
Male	107
Female	49
Side, *n* (left/right)	92/64
ASA score (*n*)	
1	56
2	93
3	7
Comorbidities (*n*)	59
Hypertension	47
Diabetes mellitus	12
Preoperative positive urine culture (*n*)	25
Previous medical history (*n*)	34
Lithotripsy surgery	24
Abdominal surgery	7
Abdominal trauma	3
Stone diameter, mm (mean ± SD)	15.6 ± 4.6
Stone CT numerical value, Hu (mean ± SD)	1,086.2 ± 322.6
Operative time, minutes (mean ± SD)	43.8 ± 25.7
Success rate of UAS placement, *n* (%)	150 (96.2)
SFR of 1 day after operation, *n* (%)	90 (57.7)
SFR of 1 month after operation, *n* (%)	137 (87.8)
Complication rate, *n* (%)	14 (9.0)
Clavien-Dindo I	9
Postoperative fever	5
Ureteral mucosal bleeding	2
Postoperative pain	2
Clavien-Dindo II	5
Urinary tract infection	5
Clavien-Dindo III	0
Clavien-Dindo IV	0
Clavien-Dindo V	0
Postoperative hospitalization time, days (mean ± SD)	2.4 ± 1.2
Preoperative creatinine, µmol/L (mean ± SD)	85.5 ± 24.1
Creatinine of 1 day after operation, µmol/L (mean ± SD)	78.1 ± 21.7
*P* value	<0.001^a^

BMI, body mass index; ASA, American society of anaesthesiologists; UAS, ureteral access sheath; SFR, stone-free rate.

a Using the student's *t* test, *P* value < 0.05 was considered an indicator of a statistically significant difference.

As shown in Table 1, stones were located on the left side in 92 cases and on the right side in 64 cases. The mean stone diameter was 15.6 ± 4.6 mm. The mean stone CT numerical value was 1,086.2 ± 322.6 Hu. The average operative time was 43.8 ± 25.7 min. The success rate of UAS placement was 96.2% (150/156). The SFR was 57.7% (90/156) at 1 day postoperatively, and 87.8% (137/156) at 1 month postoperatively. The average postoperative hospitalization time was 2.4 ± 1.2 days. Fourteen patients suffered postoperative complications, and the rate of complications was 9.0% (14/156), all of which were classified as minor (Clavien-Dindo Grades I and II). The majority of complications (9/14) were Clavien-Dindo Grade I. These included: Postoperative fever in five patients (3.2%), which was managed successfully with antipyretics (e.g., acetaminophen/paracetamol or NSAIDs) within two days post-surgery. Postoperative pain in two patients (1.3%), which was managed successfully with additional analgesic medications. Minor ureteral mucosal bleeding in two patients (1.3%), which resolved spontaneously without any intervention. The remaining complications (5/14) were Clavien-Dindo Grade II. All five cases were urinary tract infections (3.2%), all of which resolved successfully with appropriate antibiotic therapy based on bacterial culture and sensitivity results. Notably, no Clavien-Dindo Grades III-V complications occurred. Compared to the patient's preoperative creatinine levels, their creatinine levels at 1 day postoperatively were significantly improved, and the difference was statistically significant (*P* < 0.001).

### Subgroup analysis

In the subgroup analysis, as shown in Table 2, for the impacted stone group, stones were located on the left side in 50 cases and on the right side in 34 cases. The mean stone diameter was 16.6 ± 4.9 mm. The mean stone CT numerical value was 1,170.5 ± 318.1 Hu. The average operative time was 48.0 ± 29.3 min. The SFR was 54.8% (46/84) at 1 day postoperatively, and 88.1% (74/84) at 1 month postoperatively. Nine patients suffered postoperative complications, and the rate of complications was 10.7% (9/84), all of which were classified as minor (Clavien-Dindo Grades I and II). The majority of complications (5/9) were Clavien-Dindo Grade I. These included: Postoperative fever in two patients (2.4%), which was managed successfully with antipyretics (e.g., acetaminophen/paracetamol or NSAIDs) within two days post-surgery. Postoperative pain in one patient (1.2%), which was managed successfully with additional analgesic medications. Minor ureteral mucosal bleeding in two patients (2.4%), which resolved spontaneously without any intervention. The remaining complications (4/9) were Clavien-Dindo Grade II. All four cases were urinary tract infections (4.7%), all of which resolved successfully with appropriate antibiotic therapy based on bacterial culture and sensitivity results. No Clavien-Dindo Grades III-V complications occurred. Compared to the patient's preoperative creatinine levels, their creatinine levels at 1 day postoperatively were significantly improved, and the difference was statistically significant (*P* < 0.001). For the non-impacted stone group, the average operative time was 39.0 ± 19.9 min. The SFR was 61.1% (44/72) at 1 day postoperatively, and 87.5% (63/72) at 1 month postoperatively. Five patients suffered postoperative complications, and the rate of complications was 6.9% (5/72), all of which were classified as minor (Clavien-Dindo Grades I and II). The majority of complications (4/5) were Clavien-Dindo Grade I. These included: Postoperative fever in three patients (4.1%), which was managed successfully with antipyretics (e.g., acetaminophen/paracetamol or NSAIDs) within two days post-surgery. Postoperative pain in one patient (1.4%), which was managed successfully with additional analgesic medications. The remaining complications (1/5) were Clavien-Dindo Grade II. One case was urinary tract infections (1.4%), which resolved successfully with appropriate antibiotic therapy based on bacterial culture and sensitivity results. No Clavien-Dindo Grades III-V complications occurred.

**Table 2:** Subgroup analysis: preoperative calculus data and postoperative effect for impacted and non-impacted upper ureteral stones.

Variables	Impacted stone	Non-impacted stone
Patients (*n*)	84	72
Side, *n* (left/right)	50/34	42/30
Stone diameter, mm (mean ± SD)	16.6 ± 4.9	14.5 ± 4.0
Stone CT numerical value, Hu (mean ± SD)	1,170.5 ± 318.1	987.9 ± 301.2
Operative time, minutes (mean ± SD)	48.0 ± 29.3	39.0 ± 19.9
SFR of 1 day after operation, *n* (%)	46 (54.8)	44 (61.1)
SFR of 1 month after operation, *n* (%)	74 (88.1)	63 (87.5)
Complication rate, *n* (%)	9 (10.7)	5 (6.9)
Clavien-Dindo I	5	4
Postoperative fever	2	3
Ureteral mucosal bleeding	2	0
Postoperative pain	1	1
Clavien-Dindo II	4	1
Urinary tract infection	4	1
Clavien-Dindo III	0	0
Clavien-Dindo IV	0	0
Clavien-Dindo V	0	0
Preoperative creatinine, µmol/L (mean ± SD)	89.5 ± 23.5	80.9 ± 24.2
Creatinine of 1 day after operation, µmol/L (mean ± SD)	81.3 ± 22.4	74.3 ± 20.3
*P* value	<0.001^a^	<0.001^a^

SFR, stone-free rate.

^a^ Using the student's *t* test, *P* value < 0.05 was considered an indicator of a statistically significant difference.

## Discussion

Currently, percutaneous nephrolithotomy (PCNL) and URS are the primary surgical modalities for larger upper ureteral stones (19, 20). While PCNL achieves an 85%–100% success rate for treating larger upper ureteral stones (21), it is a traumatic endoscopic procedure associated with severe complications, including unbearable pain, hemorrhage (with reported bleeding rates of 14%–24%), and potential injury to adjacent organs (22–24). In contrast, URS, a minimally invasive approach utilizing the natural urinary lumen, offers the advantages of reduced trauma and quicker recovery and is a safe and effective endoscopic surgery for the treatment of ureteral calculi (25, 26). However, the application of conventional R-URS for upper ureteral stones is limited by a propensity for stone migration upward into the renal pelvis, often necessitating adjunctive measures such as stone blockers or F-URS, thereby increasing procedural time and cost (27). Although F-URS is an established first-line treatment for proximal ureteral stones less than 20 mm (9), its efficacy diminishes with stones larger than 20 mm, where it is associated with high stone retention rate and higher risks of complications, including serious infections and stone bit formation (13). Furthermore, F-URS is still expensive, less durable, and costly to maintain.

An extra vacuum suction device is the primary distinction between the novel vacuum suction semirigid ureteroscopy and the conventional ureteroscopy. The core mechanism of the vacuum suction device mainly consists of two parts: the negative pressure suction pump and the closed circulation working channel. The vacuum suction device has a perfusion interface and a suction interface. One side of the perfusion interface is connected to the perfusion fluid, and the other side of the perfusion interface, as well as the suction interface, is connected to the end of the UAS. The procedure described above creates a closed circulation working channel between the vacuum suction device and the collection system, allowing the external perfusion fluid to be supplied to the surgical area. Continuous and steady negative pressure is produced by the negative pressure suction pump and can be delivered to the surgical area via the previously mentioned closed circulation working channel. The use of vacuum suction can remove larger stone fragments and dust in time, lowering the pressure on the renal pelvis and enhancing the visibility of the surgical field. Based on the above characteristics of the vacuum suction device, the novel vacuum suction semirigid ureteroscopy presents a promising theoretical solution for larger upper ureteral stones and impacted calculi, potentially offering high clearance efficiency with minimal invasiveness. However, robust clinical evidence validating this potential remains scarce. Therefore, this study aimed to retrospectively evaluate the clinical efficacy of the novel vacuum suction semirigid ureteroscopy in treating upper ureteral stones larger than 10 mm and impacted calculi and further assess the efficacy by using a vacuum suction device during lithotripsy procedures.

**Figure 3:**
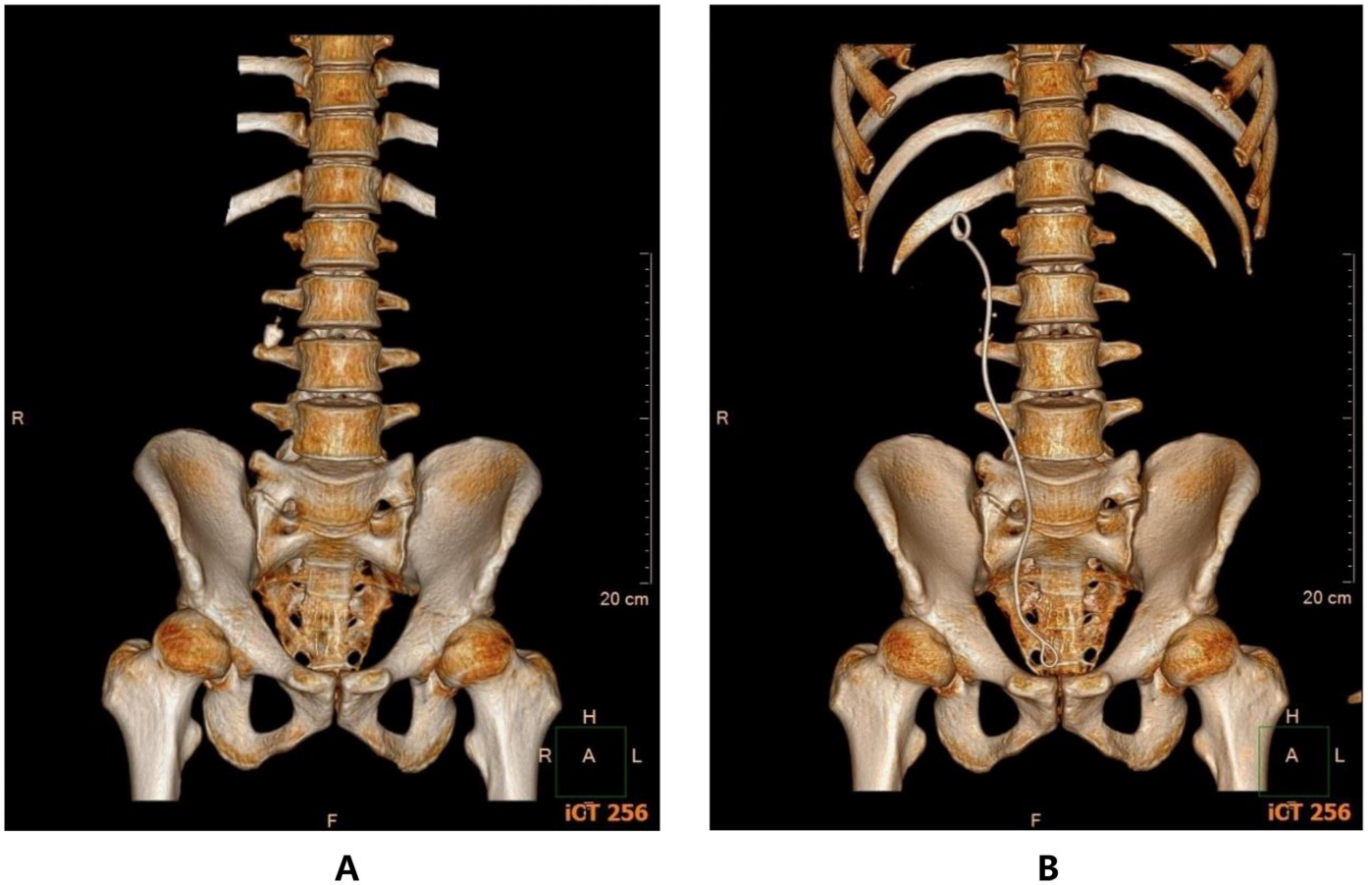
Comparison of patients' CT 3D reconstructed images: **(A)** pre-operation. **(B)** post-operation.

A study reported that semi-rigid ureteroscopy for proximal ureteral stones had an average operative time of 51.03 min and an SFR of 78% (28). In addition, a study reported that flexible ureteroscopy for proximal ureteral stones had an average operative time of 60.3 min and an SFR of 95% (9). This study showed that the novel vacuum suction semirigid ureteroscopy for >10 mm upper ureteral stones had an average operative time of 43.8 min and an SFR at 1 month postoperatively of 87.8%. In addition, this study revealed that the novel vacuum suction semirigid ureteroscopy for >10 mm impacted calculus had an average operative time of 48.0 min and an SFR at 1 month postoperatively of 88.1%. According to the study's findings, the novel vacuum suction semirigid ureteroscopy seems to be able to achieve good stone clearance outcomes and efficiency for the treatment of >10 mm upper ureteral stones and impacted calculus(Figure 3). This efficacy is primarily attributable to the special negative pressure suction device, which can remove larger stones concurrently with lithotripsy, lessen leftover stone fragments, and reduce stone escape. These elements increase the efficiency and SFR while also reducing operative time. By modifying the pressure valve of the negative pressure suction, the intraoperative pressure in the ureter and renal pelvis can be regulated, thereby decreasing the likelihood of stone escape, the rate of stone retention, and the risk of stone bit formation following surgery. Furthermore, the negative pressure suction effect can draw out the air bubbles, blood clots, and gravel created during the stone crushing process, making the surgical field of view clearer, reducing operative time, and increasing stone removal efficiency.

**Figure 4:**
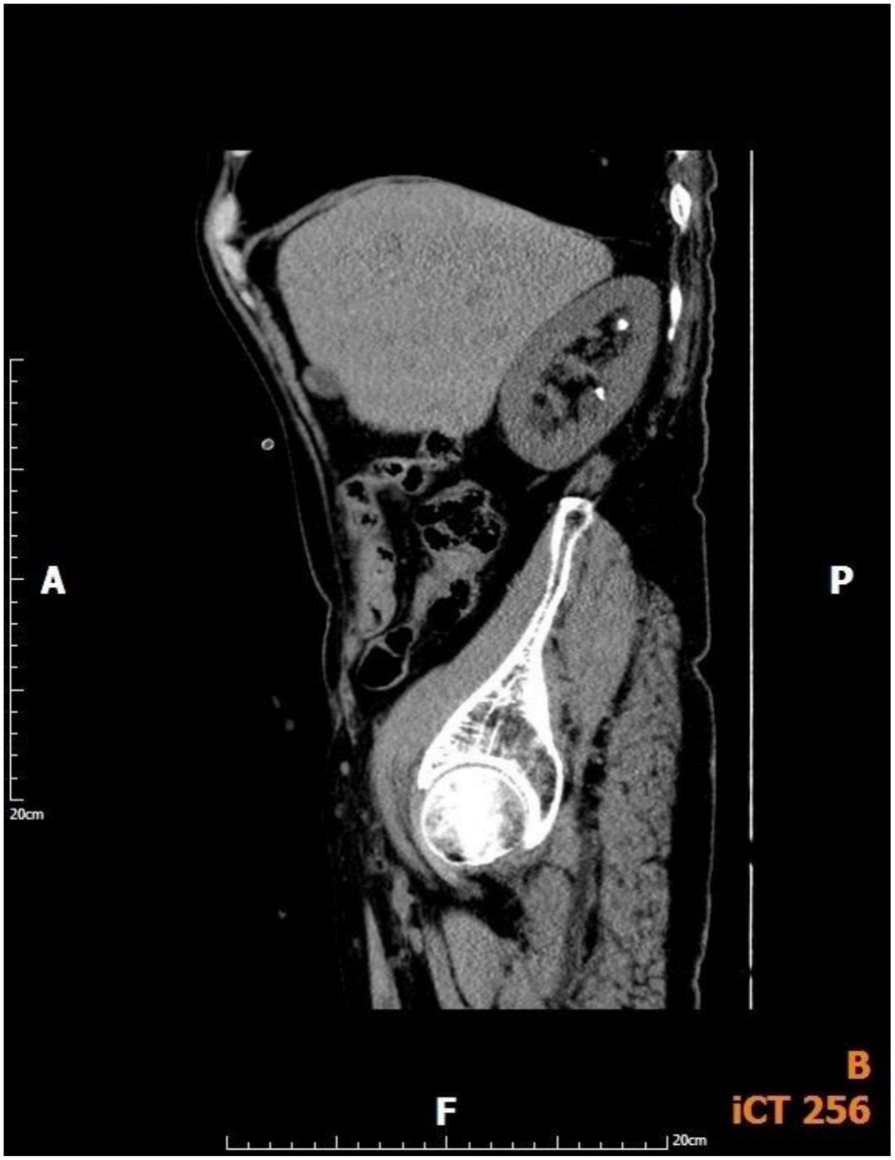
CT images of patient with incomplete stone removal on the first day after surgery.

In this study, the SFR at 1 day postoperatively was 57.7% for >10 mm upper ureteral stones and 54.8% for impacted calculi, which appears suboptimal. This can be explained by two main factors. First, the study cohort involved larger (mean diameter >15 mm) and impacted stones. The laser fiber may find it difficult to reach the stone's core if it is heavily impacted or encircled by granulation tissue. This could lead to low gravel efficiency and the generation of sizable, difficult-to-evacuate fragments. Second, despite the suction capability, the surgeon must be skilled in the dynamic balance of negative pressure suction and perfusion flow. Inadequate suction timing or pressure, or suction tubing obstruction by larger fragments, could cause fragments to escape beyond the negative pressure suction range into the renal calyx and remain within the kidney. Postoperative imaging confirmed that most residual fragments were located intrarenal (Figure 4). While combined use with F-URS could manage such migrated fragments, this study only included cases that were successfully completed using the novel vacuum suction semirigid ureteroscopy alone.

A study reported that the rate of postoperative complications of patients with proximal ureteral stones treated with semirigid ureteroscopy was 16% (28). In addition, a study reported that the rate of postoperative complications of patients with proximal ureteral stones treated with flexible ureteroscopy was 8.4% (9). This study showed that the novel vacuum suction semirigid ureteroscopy had a rate of postoperative complications of 9.0% for >10 mm upper ureteral stones and 10.7% for impacted calculus. According to the study's findings, the novel vacuum suction semirigid ureteroscopy seems to be able to achieve safe surgical outcomes, which may be related to the fact that the novel vacuum suction semirigid ureteroscopy has a special negative pressure suction device. By adjusting the negative pressure suction device, the surgeon can actively control the suction force, maintaining low pressure in the renal pelvis. These maneuvers may reduce the incidence of complications such as bleeding, infection, and septic shock caused by elevated renal pelvis pressure. Continuous convection water circulation created by negative pressure suction can quickly remove heat produced by the holmium laser lithotripsy procedure (29), reduce ureter thermal damage, and lower the risk of surgical complications like ureteral injuries, avulsions, and even perforation.

The novel vacuum suction semirigid ureteroscopy is a surgical technique that combines negative pressure suction technology. While it can produce secure and effective surgical results in clinical practice, its use is still fraught with issues related to cost, technical accessibility and security. Firstly, compared with traditional endoscopes, it requires specialized UAS, a vacuum suction device, and ancillary equipment, resulting in higher overall procurement costs. Vacuum suction devices require regular maintenance, and UAS are worn out and replaced more frequently (especially when dealing with complex stones), leading to higher maintenance costs. Secondly, it is highly dependent on vacuum suction devices, perfusion equipment, and ancillary facilities and may be difficult to popularize in areas where medical resources are scarce (e.g., in some developing countries). Thirdly, it requires the physician to be skilled in the dynamic balance of negative pressure suction and perfusion flow, and the learning curve may be longer than that of conventional endoscopy. In addition, the rigid ureteral sheath is made of metal, and the placement of which in combination with rigid instruments may increase the risk of ureteral injury. There is a theoretically higher risk of ureteral injury for patients with large impacted ureteral stones (>10 mm) that have been in place for over two months. Such stones often induce mucosal inflammation, granulation tissue formation, edema, and fibrosis, leading to ureteral stiffness and luminal narrowing (30). Advancing the abovementioned rigid combination through narrowed or tortuous segments increases the potential for mucosal stripping, deep laceration, or even perforation. Therefore, surgeons must possess detailed anatomical knowledge and employ a “gentle and mild” technique during the insertion of the rigid ureteral sheath combination, particularly at sites of ureteral physiological narrowing.

Furthermore, a critical consideration with this novel ureteroscopy is the management of intrarenal pressure (IRP). While the vacuum suction can lower IRP, the absence of integrated pressure monitoring poses a potential risk (31). Procedures for larger and impacted stones often require prolonged laser use and higher irrigation flows to maintain visibility, which can elevate IRP and increase the risk of postoperative fever and infection (32). Furthermore, excessive suction force itself may theoretically cause ureteral mucosal bleeding or injury, a concern supported by two observed cases of minor mucosal bleeding in this study. Therefore, surgical success and safety are highly dependent on the surgeon's ability to manually maintain a dynamic balance between suction and irrigation. The current lack of integrated pressure monitoring and control devices represents a primary limitation of this novel ureteroscopy (31). Future technological iterations could significantly benefit from incorporating intelligent pressure-control systems, such as the recently reported intelligent platform that maintains a safe, real-time monitored renal pelvic pressure during F-URS (33–37). Integrating such advancements would be a crucial step forward in standardizing the safety profile of the novel vacuum suction semirigid ureteroscopy. In addition, the flexible navigable suction ureteral access sheath (FANS) represents a significant advancement in sheath technology recently, integrating continuous suction with a flexible, navigable tip (38, 39). In contrast to the rigid UAS used in our study, FANS can better conform to the natural anatomy of the upper urinary tract, potentially improving the capability to reach the renal pelvis and calyces and reducing the risk of iatrogenic injury. Its successful application in treating larger stones (20–30 mm) highlights the clinical value of combining suction with excellent flexibility (40, 41). The evolution of the novel vacuum suction semirigid ureteroscopy could draw valuable insights from these technologies in the future. Incorporating the intelligent pressure-control systems and flexible, navigable sheath designs would be a critical step toward developing next-generation devices that are both more efficient and safer.

This study provides an initial investigation into the clinical efficacy of the novel vacuum suction semirigid ureteroscopy in treating >10 mm upper ureteral stones and impacted calculus. Additionally, it offers an initial evaluation of the efficacy of utilizing a vacuum suction device during lithotripsy procedures. However, this research has certain limitations, including a lack of data on other lithotripsy procedures as a control, a small sample size, and a non-randomized retrospective design, as well as data sourced from a single institution. In the future, a carefully designed prospective study involving a large-sample, multicenter, randomized, and controlled study is required to further validate the current findings.

## Conclusion

The novel semirigid ureteroscopy with a vacuum suction device is a safe and effective surgical procedure for the treatment of >10 mm upper ureteral stones and impacted calculus. During the lithotripsy procedure, the use of a vacuum suction device has the potential to improve stone removal efficiency. However, due to the critical limitation of the lack of integrated pressure monitoring and control devices, potential complications caused by negative pressure suction and perfusion flow imbalance should be watched out for.

## Data Availability

The raw data supporting the conclusions of this article will be made available by the authors, without undue reservation.
